# Efficacy of Essential Oils in Relieving Cancer Pain: A Systematic Review and Meta-Analysis

**DOI:** 10.3390/ijms24087085

**Published:** 2023-04-11

**Authors:** Maria Tiziana Corasaniti, Giacinto Bagetta, Luigi Antonio Morrone, Paolo Tonin, Kengo Hamamura, Takafumi Hayashi, Francesca Guida, Sabatino Maione, Damiana Scuteri

**Affiliations:** 1 Department of Health Sciences, University “Magna Graecia” of Catanzaro, 88100 Catanzaro, Italy; 2 Pharmacotechnology Documentation & Transfer Unit, Department of Pharmacy, Preclinical & Translational Pharmacology, Health & Nutritional Sciences, University of Calabria, 87036 Rende, Italy; 3 Regional Center for Serious Brain Injuries, S. Anna Institute, 88900 Crotone, Italy; 4 Department of Clinical Pharmacokinetics, Faculty of Pharmaceutical Sciences, Kyushu University, Fukuoka 812-8582, Japan; 5 Laboratory of Pharmaceutical Sciences, Faculty of Pharmaceutical Sciences, Tohoku Medical and Pharmaceutical University, Sendai 981-8558, Japan; 6 Department of Experimental Medicine, Division of Pharmacology, University of Campania “L. Vanvitelli”, 80138 Naples, Italy; 7 IRCSS, Neuromed, 86077 Pozzilli, Italy

**Keywords:** cancer pain, oncologic pain, tumor pain, essential oils, aromatherapy, integrative medicine

## Abstract

Over 80% of patients affected by cancer develops cancer-related pain, one of the most feared consequences because of its intractable nature, particularly in the terminal stage of the disease. Recent evidence-based recommendations on integrative medicine for the management of cancer pain underline the role of natural products. The present systematic review and meta-analysis aims at appraising for the first time the efficacy of aromatherapy in cancer pain in clinical studies with different design according to the most updated Preferred Reporting Items for Systematic reviews and Meta-Analyses (PRISMA) 2020 recommendations. The search retrieves 1002 total records. Twelve studies are included and six are eligible for meta-analysis. The present study demonstrates significant efficacy of the use of essential oils in the reduction of the intensity of pain associated with cancer (*p* < 0.00001), highlighting the need for earlier, more homogeneous, and appropriately designed clinical trials. Good certainty body of evidence is needed for effective and safe management of cancer-related pain using essential oils by establishment of a step-by-step preclinical-to-clinical pathway to provide a rational basis for clinical use in integrative oncology. PROSPERO registration: CRD42023393182.

## 1. Introduction

The term “cancer pain” best characterizes the multidimensional and multifaceted nature of pain, since it includes physical, psychosocial, and quality of life domains, often co-occurring with the so-called cancer symptoms cluster including anxiety, depression, and sleep disturbances [1,2]. The issue posed by cancer pain, due to surgical and chemotherapy treatment alongside the tumor-specific features, is a matter of urgency due to its tight link with reduction of the overall quality of life and the increasing number of cancer-affected patients. In fact, the improvement of early detection systems and global aging cause a continuous increase in cancer survivors, with an estimated 18 million people with a history of cancer up to the beginning of 2022 in the United States [3]. All the cancer symptom clusters may share common biologic bases, likely due to the involvement of intense and smoldering inflammatory and immune responses elicited by the cancer pathogenesis environment, but also by treatment [4].

Over 70–80% of patients affected by tumors is reported to suffer from cancer pain in meta-analyses concerned with cancer pain prevalence in a 40 year-period [5,6]; this pain is unbearable in up to 33% of patients [7] and reaches the 95% rate in patients affected by advanced disease (see [8]), making cancer pain a major problem in the management of oncologic patients. Moreover, the latest systematic review dealing with pain prevalence in cancer survivors dates back to 2022 [9]: it underlines that pain prevalence in solid tumor survivors that had finished treatment at least 3 months earlier is 47%, with a heterogeneity of 98.99% among studies. These data support the importance of cancer pain treatment for the management and the improvement of quality of life of cancer patients.

In addition, cancer pain is often persistent and chronic [10], with breakthrough characteristics. It represents one of the most feared consequences of cancer due to its intractable nature in the terminal stage of the disease, causing about 25% of patients to die in significant pain (see [8]).

Chronic pain, often including inflammatory and neuropathic features as they occur in cancer pain, is one of the most common reasons to arrive at clinical observation [11]. Cancer pain includes syndromes related to surgical or chemotherapy/radiation/hormonal therapy treatments [12]. In fact, neuropathic pain, due to a lesion or disease of the somatosensory system [13], can occur after surgery procedures [14] or injury [15] and also stroke [16], but also to neuropathies and headache [17] resulting from chemotherapeutic treatments causing chemotherapy-induced peripheral neuropathy (CIPN), which is often under-recognized and under-treated [18,19].

Furthermore, the treatment of chronic pain in aged patients is complicated by: the lack of information about appropriate use and dose of analgesics due to exclusion of these patients from clinical trials [20], particularly for anti-migraine treatments [21,22,23]; polypharmacy [24]; physiological differences [25,26]. This lack of information is due to the practice of excluding aged patients. The use of essential oils endowed with proven analgesic properties can be the safest option in the frame of integrative medicine in oncology.

A recent guideline (2022), produced to provide clinicians with evidence-based recommendations about integrative approaches for the management of pain in cancer patients, reports the role of natural products [27]. In particular, aromatherapy is found to have low levels of evidence for CIPN treatment, having reported only five randomized, controlled trials [27]. A previous systematic review assessing the efficacy of complementary and alternative therapies in cancer pain had already highlighted the low quality of research on herbal supplements and the scarcity of randomized, controlled trials in this field [28].

Preclinical research from our group built the rationale for clinical translation of the essential oil of bergamot (BEO) for continuous administration as aromatherapy, and for transdermal application of an engineered, nanotechnological, pharmaceutical form named NanoBEO released by an airless dispenser [29,30,31,32,33,34,35,36,37]. Since BEO proved its analgesic effectiveness both in inflammatory and in neuropathic pain models, reliable to clinic, it could represent a candidate for the treatment of cancer pain.

The aim of the present systematic review and meta-analysis is to appraise for the first time the efficacy of aromatherapy in the reduction of cancer pain through clinical studies of any design, following the most updated Preferred Reporting Items for Systematic reviews and Meta-Analyses (PRISMA) 2020 recommendations [38]. Moreover, information concerned with the type of essential oils used and effectiveness can offer points for reflection about the possible mechanisms involved in cancer pain control. The present systematic review is registered in the National Institute for Health Research (NIHR) International prospective register of systematic reviews (PROSPERO) with number CRD42023393182.

## 2. Materials and Methods

### 2.1. Objectives and Protocol

The search, extraction, and selection of the results is carried out in agreement with the most recently updated Preferred Reporting Items for Systematic reviews and Meta-Analyses (PRISMA) 2020 recommendations [38,38,39,40]. The research question is formulated as a PICOS (participants/population, interventions, comparisons, outcomes, and study design) question. The participants are patients affected by cancer pain. The intervention consists of essential oils administered in any dose and route. Studies are deemed to be eligible if they assess the effectiveness of the intervention over the comparator consisting in placebo/no treatment or active control, i.e., any drug approved for pain treatment. Study designs to be included are prospective and retrospective clinical studies. The primary outcome consists in pain reduction. In vivo and in vitro preclinical studies, reviews, book chapters, and congress communications and proceedings are excluded. The protocol was established prior to the literature search and registered in PROSPERO (CRD42023393182). The titles and the abstracts and subsequently the full text of the retrieved studies, are screened by two independent review committee members based on the a priori established inclusion and exclusion criteria. The references of the most significant papers are inspected to avoid missing of additional studies. Any disagreement is solved by achieving consensus through the Delphi method [41] or by consulting a third team member.

### 2.2. Information Sources

PubMed/MEDLINE, Scopus, and WOS are inspected for peer-reviewed studies published from database inception to the present without date restriction up to the date of last search on 3 February 2023. Only articles published in English are included. After title and abstract screening, articles not available in full text are excluded. Two independent members of the review committee search for records matching the strategy strings.

### 2.3. Search Strategy

The following medical and subject headings (MeSH) terms are used in combination within search strings: “Cancer”; “Oncologic”; “Tumo(u)r”; “Pain”; “Essential oils”; “Aromatherapy”; “Integrative medicine”. PubMed/MEDLINE are searched for MeSH terms, Scopus for Article Title, Abstract, Keywords and WOS for all fields. The search has the characteristics of a high sensitivity/recall search strategy, keeping precision [42]. The lines and spelling of search strings, the capability of the search to cover all the most relevant aspects and the accuracy to answer to the PICOS question are checked by an author different (reviewer) from the two searching the databases independently (requestors) in agreement with the evidence-based guideline for Peer Review of Electronic Search Strategies (PRESS) for systematic reviews (SRs) [42,43].

### 2.4. Study Selection

Two authors independently assess the eligibility of the retrieved results, in order to minimize the risk to miss relevant records. Duplicates are removed by reference manager software (EndNote X7, Clarivate, London, UK). Title and abstract and subsequently full text are screened.

### 2.5. Data Synthesis, Assessment of the Risk of Bias and Critical Appraisal

The synthesis of the results is performed according to the Cochrane Consumers and Communication Review Group guidelines [44]. The risk of bias (RoB) and the quality/certainty [45] of the body of evidence are assessed independently by two members of the review committee according to PRISMA 2020 statement [46] using: the revised Cochrane risk of bias tool RoB2 for randomized clinical trials [47]; the ROBINS-I tool [48] for studies not randomized. The visualization of the risk of bias assessment is produced with the Cochrane robvis visualization tool [49].

### 2.6. Statistical Analysis and Effect Measures

The Cochrane Review Manager 5.4.1 (RevMan5.4.1; Copenhagen: The Nordic Cochrane Center, The Cochrane Collaboration) is used to measure and 95% confidence intervals (CI) or standardized mean differences (SMD) and inverse variance for dichotomous and continuous variables, respectively. The paucity of studies eligible for the analysis does not allow to plan a sensitivity analysis, restricting the primary analysis to low-risk-of-bias studies, or following subgroup analysis or meta-regression based on stratification of the studies. The heterogeneity of the retrieved results is calculated through the random effect model [50] and the Higgins I^2^ value [51], while Egger’s linear regression test [52] for funnel plot asymmetry [53], adjusted through the “trim and fill” method [54], is used to evaluate the publication bias.

## 3. Results

### 3.1. Studies Selection

**Figure 1:**
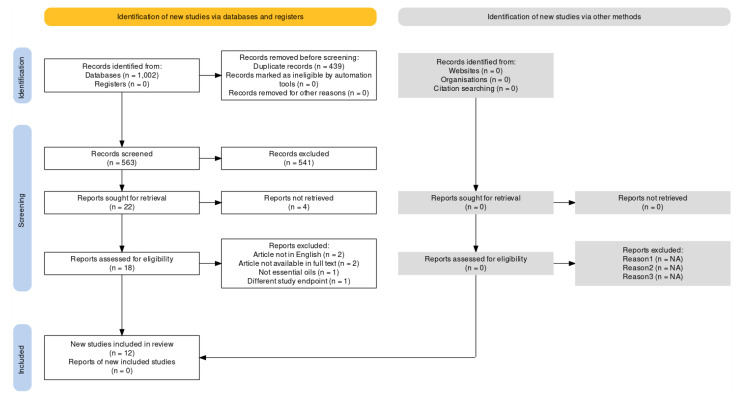
Process of extraction of results according to the Preferred Reporting Items for Systematic Reviews and Meta-Analyses (PRISMA) 2020 flow diagram produced with the web-based Shiny app [70].

The databases searched are PubMed/MEDLINE, Scopus, and Web of Science (WOS) since their inception until the date of last search, i.e., 3 February 2023. The search retrieves 1002 total records. In particular, 131 records are retrieved from PubMed/MEDLINE, 563 from Scopus and 308 from WOS. No additional studies are identified by reference list screening. Duplicate removal leads to 563 studies remaining. The title and abstract screening leads to the exclusion of records not meeting the inclusion criteria (for different outcomes investigated or study design, e.g., in vitro and in vivo preclinical studies, case reports, qualitative clinical studies, surveys, reviews, book chapters, congress abstracts, proceedings, etc.), and also for the intervention used. In fact, some records might appear to meet the inclusion criteria, but the intervention consists of extracts or essential oil components, and not essential oils or aromatherapy. For instance, the study by Hasheminasab et al., 2020 [55] is excluded because the herbal treatment used is not an essential oil. In addition, in the records of Aghamohammadi and collaborators (2018) [56] and of Arantes et al., 2021 [57], the intervention contains an extract, not an essential oil. The studies of Czakert [58] and of Ho [59] and coworkers are excluded because they perform a qualitative analysis. After title and abstract screening, 22 records are left. In particular, the papers by Oyston and McGee, 2012 [60], by Lee and Park, 2018 [61], and by Cheong et al., 2022 [62] are not available in full text; therefore, they are excluded. The articles of Xiao and collaborators (2018) [63] and of Chang, 2008 [64] are excluded due to being in Chinese. The records of Nekuzad et al., 2012 [65] and the quasi-randomized, controlled, pilot study conducted by Yayla and Ozdemir in 2019 [66] are not eligible because the articles are not available in full text. The study of Corbin et al., 2009 [67] is not eligible for inclusion in the analysis since it is a letter to the editor reporting about the use of complementary and alternative medicine, but not aromatherapy or essential oils specifically. The paper by Ovayolu et al., 2014 [68] cannot be included in the analysis because pain is not an endpoint of the study. The study of Elhadad and colleagues (2022) [69] cannot be included because the intervention is based on a gel of chamomile alcoholic extract. Full text screening leaves 12 results to be included in the analysis. The process of extraction of the studies is illustrated in Figure 1.

### 3.2. Data Synthesis, Critical Appraisal, and Meta-Analysis

**Figure 2:**
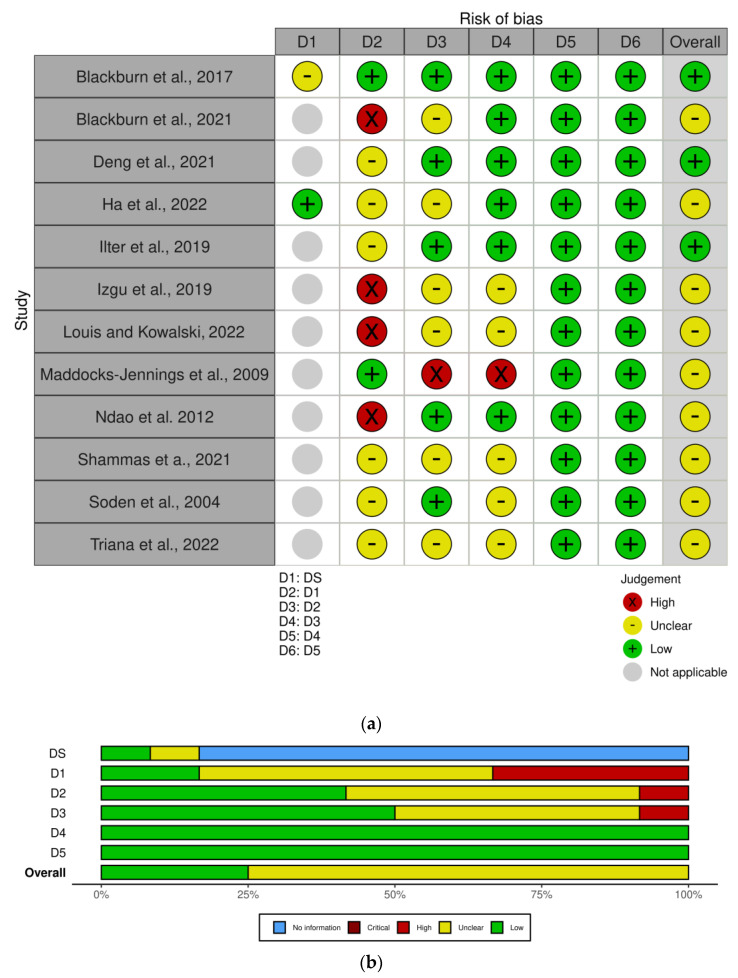
Risk of Bias assessment as traffic-light plot (**a**) and weighted bar plots (**b**). The visualization of the risk of bias assessment is produced with the Cochrane robvis visualization tool [49,71,72,73,74,75,76,77,78,79,80,81,82], and evaluated according to the revised Cochrane risk of bias tool RoB2 for randomized clinical trials and the Risk of Bias in Non-randomized Studies—of Interventions (ROBINS-I) tool for studies not randomized.

The study by Blackburn et al., 2017 [71] is a randomized, cross-over, wash-out trial (design chosen to keep into account the inter-individual variability to therapy-induced symptoms) in which 50 patients with acute, myelogenous leukemia subjected to chemotherapy receive aromatherapy. Lavender is the scent selected most often in this study, followed by peppermint and then chamomile. Randomization uses a computer-generated table and baseline characteristics do not suggest concern; therefore, there is no risk of bias in terms of domain 1, i.e., randomization, using the RoB 2 tool for cross-over trials according to the situation when data from both periods are analyzed appropriately, accounting for the pairing of observations across the two periods for each individual. In the first week, patients are randomized to placebo or aromatherapy, followed by wash-out in the second week, and then placebo or aromatherapy in the third week. Twenty-five patients undergo aromatherapy first and 28 patients undergo placebo first. However, some bias arising from carry-over effects (domain S) is recorded because intervention-by-time period interactions should be included. Only three patients do not receive interventions as planned; therefore, a number under 10% of the total sample, that is, the domain 2 of deviations from intended intervention can be deemed unbiased. Out of the fifty-three patients enrolled, 50 complete the study and 53 patients demonstrate 91.8% power according to sample power calculation; therefore, there is no concern in terms of bias due to missing outcome data (domain 3). In addition, no bias in measurement of the outcome (domain 4) is detected, since the measurement systems of the outcome are appropriate using the Edmonton Symptom Assessment Scale–Revised with Visual Analog Scale (VAS). No risk of bias in selection of the reported results (domain 5) appears. Bias assessment is reported in Figure 2. In this study, aromatherapy affords improvement of tiredness, drowsiness, lack of appetite, depression, anxiety, and well-being, but not a reduction of pain.

The study by Blackburn and collaborators performed in 2021 [72] involves patients affected by advanced cervical cancer undergoing brachytherapy for administration of high doses of radiation to the primary tumor and sparing doses to the organs at risk. This procedure is invasive, causing pain and anxiety. Therefore, this randomized, controlled trial aims at assessing the efficacy of aromatherapy with lavender, lemon, or peppermint and foot reflexology to reduce pain and anxiety in patients receiving brachytherapy for cervical cancer. Even without statistical analysis, as in the study of Blackburn and coworkers of 2017, the baseline characteristics are not suggestive of bias for pain assessment, apart from what occurs for the item accounting for opioid use prior to the study, used by a median of nine patients in the intervention group and by a median of four in the control group (20% difference, as reported by the Authors). This issue is managed through the within-subject repeated measures, to account for the possible correlation of pain scores from the same subject. Therefore, although patients are randomized using GraphPad software, concern in terms of randomization bias is reported (domain 1). The sample of 41 patients fulfils the sample size calculation performed, according to which the minimum sample size was set at 40 patients to achieve at least 80% power. Therefore, in spite of the six participants who were unable to complete the data for the full five fractions of treatment, there is some risk of bias in domain 2, but no concern in terms of bias due to missing outcome data (domain 3). Pain is measured using a numeric rating scale (NRS). Since the measurement system consisting in the NRS is appropriate, no risk of bias in measurement of the outcome (domain 4) is reported. No risk of bias in selection of the reported results (domain 5) is signaled. The effect of the intervention, after reflexology, is statistically significant (*p* < 0.0001).

The study conducted by Deng et al., 2021 [73] deals with the effect of perioperative aromatherapy, with or without music therapy, on pain and anxiety levels in women after breast cancer surgery. A total of 160 patients with breast cancer are randomly assigned in a 1:1:1:1 ratio to receive usual care, aromatherapy, music therapy, or combination therapy during perioperative periods. Pain measure consists of VAS; thus, no risk of bias in measurement of the outcome (domain 4) is reported. Interestingly, interleukin (IL) 6 levels are assessed as biomarker. The open-label nature of the study, without blinding, raises some concern in terms of risk of bias for allocation (domain 1). On the other side, differences in baseline characteristics are compared by χ^2^ tests for categorical variables, showing no significant difference in demographic and clinical characteristics at baseline. A number of 196 patients is screened for eligibility, and 160 participants are randomly allocated to the protocol: although the number of patients is high for aromatherapy studies, no sample power calculation is reported. Data do not suggest bias in domain 2, 3, 4, and 5. According to the results, 4 h after tracheal extubation following surgery, combination therapy is the most effective option, and it is more effective than aromatherapy alone; these results are paralleled by IL-6 level reduction. Unfortunately, the choice of essential oil is an important factor that is not explored in the present study.

The research performed by Ha et al., 2022 [74] consists of a phase II, randomized, cross-over trial that aims to assess the effects of aroma lymphatic tressage on pain in breast cancer patients who are going to receive taxane-based chemotherapy. Frankincense is used as essential oil and sweet almond oil as a carrier oil. The aroma lymphatic tressage is applied in addition to standard care for pain, in comparison with standard care only, on 4 consecutive days from the day after taxane administration. Baseline differences in age raise some concerns, in spite of automatic randomization and allocation. There is a period of two weeks of wash-out, important in cross-over clinical trials, and, in appropriate manner, period/cycle evaluations are also reported separately for cycle 1, assuming the absence of carry-over effect, as stated by the Authors (low bias in domain S). Moreover, treatment intervention effect and period effect between cycles 1 and 2 are calculated. To obtain 80% power with a 5% significance level, 37 patients in each group, therefore a total of 74 patients, are enrolled to the study. During the study, nine patients refuse to continue the intervention; thus, 65 patients complete the trial per protocol. The outcome measure is recorded through VAS. Accordingly, no risk of bias in measurement of the outcome (domain 4) is reported. In addition, no risk of bias in selection of the reported results (domain 5) is detected. Peak pain scores between treatment options in both cycles show 5.05 ± 2.56 for intervention and 5.28 ± 2.45 for standard care. Therefore, no significant difference in pain score (*p* = 0.368) or toxicity is reported.

The study by Ilter et al., 2019 [75] is a non randomized, controlled trial that evaluates the effect of inhaler aromatherapy on invasive pain during port catheter insertion in cancer patients. Therefore, there are some concerns in terms of randomization bias, although the baseline characteristics of patients are similar (*p* > 0.05), except for educational level (*p* = 0.047). Aromatic mixture is obtained by diluting orange, chamomile, and lavender oil at the ratio of 1:1:1 in 70 mL distilled water. According to the sample power calculation, the minimum number of patients required is 17 and the trial is completed by 30 patients for the intervention group and 30 patients for the control group. Furthermore, no patient requests intervention interruption or decides not to continue the study. It is important to consider that control group assessments are conducted appropriately before the intervention group to prevent control group participants being exposed to any aromatherapy residual. In addition, vital signs before, during, and after the procedure and the procedure adherence are assessed. The effect of aromatherapy reduces pain during and after the procedure in a statistically significant manner in comparison with control (i.e., no treatment).

The study by Izgu et al., 2019 [76] is an open-label, parallel-group, quasi-randomized (stratified randomization), controlled, pilot study, raising a high risk of bias in terms of randomization and allocation. This study aims at investigating the effect of aromatherapy massage on CIPN pain in cancer patients treated with oxaliplatin. It is noteworthy that the Authors report that a placebo control is not performed because of the characteristic odor of the essential oil blend (peppermint, chamomile, and rosemary blended in 1:1:1 proportion at 1.5% in 50 mL of coconut oil), hence using routine care as comparator. It is reported that the essential oil blend is stored in lightproof and airtight 50 mL glass bottles. Appropriately, neuropathic pain is assessed using the Douleur Neuropathique 4 Questions (DN4), without risk of bias in domain 4. According to sample power calculation performed using software package G*power, version 3.1.7, N = 22 is enough for each group of this study. Forty-six patients are quasi-randomly assigned to the intervention (N = 22) and to the control (N = 24) groups, similar in terms of baseline characteristics (*p* = 0.627). During the study period, in the intervention group two patients drop out and in the control group, four patients drop out and two discontinue. In both groups, one patient undergoes deviations from intended protocol due to infection. At week 6, the rate of neuropathic pain is significantly lower in the aromatherapy massage group (N = 4, % = 18.2; N = 11, % = 45.8; *p* = 0.046). This study cannot be included in the meta-analysis since the assessment is too heterogeneous (DN4, with dichotomous variables) to be compared with the other studies.

The study of Louis and Kowalski of 2022 [77] assesses the effectiveness of aromatherapy with humidified lavender essential oil on pain, anxiety, depression, and sense of wellbeing in 17 cancer patients. The design of this study is a quasi-experimental, repeated-measures, one-group design; therefore, the risk of bias is evaluated according to the ROBINS-I tool. The baseline characteristics table with statistical analysis is not reported, thus raising concern in terms of risk of bias due to confounding factors and the selection of participants to the study, grouped under domain 1 to allow comparison with the other randomized studies. Since the scale selected is VAS, there is no risk of bias in measurement of outcome. No sample size or mention of drop-out or missing data are reported. Pain decreases from 1.70–1.66 of control groups to 1.25 of intervention. This study cannot be included in the meta-analysis since the standard deviation is not reported.

The study of Maddocks-Jennings and coworkers of 2009 [78] is a randomized, placebo-controlled, feasibility study investigating the effect of gargle containing two drops of a 1:1 mix of the essential oils of manuka and kanuka in water on radiation-induced mucositis of the oropharyngeal area during treatment for head and neck cancers. As reported by the Authors, a significant limitation of this study is the small sample size (N = 19). A convenience sample of twenty-six patients is chosen but four patients drop out voluntarily, two of the active group discontinue, and one patient in the control group deviates from intended intervention. Due to the small sample size, these issues raise a high concern of risk of bias, but clinical staff and radiation oncologists conducting the assessments are blind to which treatment arm patients belong to. Pain due to mucositis is appropriately measured through VAS scale. Within the active group, n = 2 patients experience pain scores ≥3, n = 5 from the control group, and n = 4 from the placebo group. This study cannot be included in the meta-analysis due to heterogeneity of outcome measure reporting.

The randomized (randomization is stratified by age), placebo-controlled, double-blind trial conducted by Ndao et al. in 2012 [79] aims at assessing the effect of inhalation of bergamot essential oil on pain, anxiety, and nausea in children and adolescents subjected to stem cell infusion. It involves 37 out of 40 patients needed to provide 80% power, but no patient drops out during the study after allocation. Baseline characteristics do not show statistically significant differences, except for baseline pain, as reported by the Authors. The choice of the placebo, consisting in non-essential-oil-based scented shampoo, is not directly comparable to the intervention. Another limitation of this study, stated by the Authors, is the small sample size consisting of patients with different diagnoses and treatment histories. The schedule of treatment is very different from the other studies, thus preventing meta-analysis, and it is as follows: within one week prior to transplantation (T1), following administration of intravenous medications and prior to stem cell or bone marrow infusion (T2), upon completion of infusion (T3), and one hour following completion of the infusion (T4). No significant effect on pain is recorded.

The study by Shammas et al., 2021 [80] is a prospective, single-blinded, randomized, controlled trial with the purpose of evaluating the effect of lavender oil (placed on left and right wrists to rub with hands, and on the temples intraoperatively) on anxiety, depression, pain, and sleep in women subjected to microvascular breast reconstruction due to breast cancer. Fractionated coconut oil is selected as control being colorless, odorless, and inert in nature. The randomization is a block randomization and only the study coordinator knows the allocation. Out of the 29 patients allocated per group, 27 in the intervention and 22 in the control group complete the trial. A limitation of the study reported by the Authors is that the sample size is not calculated a priori, but chosen arbitrarily. No significant effect on pain occurs, in the absence of any significant adverse events or complications. Since pain results are reported as a mixed model accounting for ArmxTime, these data are not suitable for meta-analysis.

In the randomized, controlled trial performed by Soden and colleagues in 2004 [81], forty-two patients are randomly allocated to aromatherapy massage with lavender essential oil and an inert carrier oil (aromatherapy group) in comparison with massage with an inert carrier oil alone (massage group) or no intervention (control group). Pain intensity is appropriately assessed through VAS and Verbal Rating Scale (VRS). In fact, the primary outcome of the present study is the effect on pain intensity scores, while the secondary outcomes include sleep quality, anxiety, depression, and overall quality of life. In addition, the sample power calculation is conducted to be able to detect an improvement in pain from the baseline of 2.3 points VAS in comparison with the control group (with a power = 80%), recruiting 15 patients to each arm of the study. Patients are randomly allocated to one of three groups, treatment allocation is concealed during baseline assessments, and the patients and the researchers recording and analyzing the data are blinded to the interventions, thus preventing risk of bias for randomization and allocation concealment. However, some concerns are raised by the evidence reported by the Authors that although no significant difference is noted in baseline assessments, also for pain intensity, there are significantly more women in the control group than either of the other groups (*p* = 0.02) and cases of depression scores cut-off threshold in massage than in aromatherapy massage group (*p* = 0.03). Six patients do not complete the study. There is no statistically significant difference in pain intensity from final to baseline assessment and, since standard deviation is not reported, this study is not eligible for meta-analysis.

Triana and collaborators in 2022 [82] perform a quasi-experimental study with a consecutive sampling technique with the purpose of understanding the effectiveness of aromatherapy in school-age children and adolescents (7–17 years) with a diagnosis of cancer, experiencing chronic pain (for longer than 1 month) and receiving moderate analgesics/opioids. A sample of 20 patients are randomly allocated to two groups: Intervention (N = 10), consisting in aromatherapy with the most favored scent (most often aloe vera) and Control (N = 10) with standard care. The small sample size and the lack of mention of any drop-outs and missing outcomes raise some concerns of risk of bias. The nurses administer an aromatherapy intervention by dripping four drops of the selected essential oil on clean gauze, sticking it to the patient’s chest at 20 cm, and assessing pain using VAS at 10 and 30 min. This study demonstrated that inhaled aromatherapy reduces chronic pain (*p* = 0.001) compared with standard care.

The main characteristics of the studies included in the analysis are reported in Table 1.

**Figure 3:**
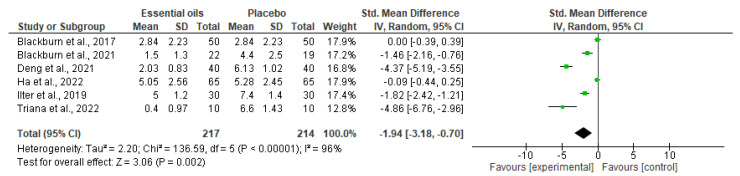
Forest plot of the meta-analysis for which six out of 12 retrieved studies are eligible. Essential oils represent the intervention, alone or in combination with other treatments, and placebo represents the control or the most inert comparator included in the studies with more than one comparator [71,72,73,74,76,82].

**Figure 4:**
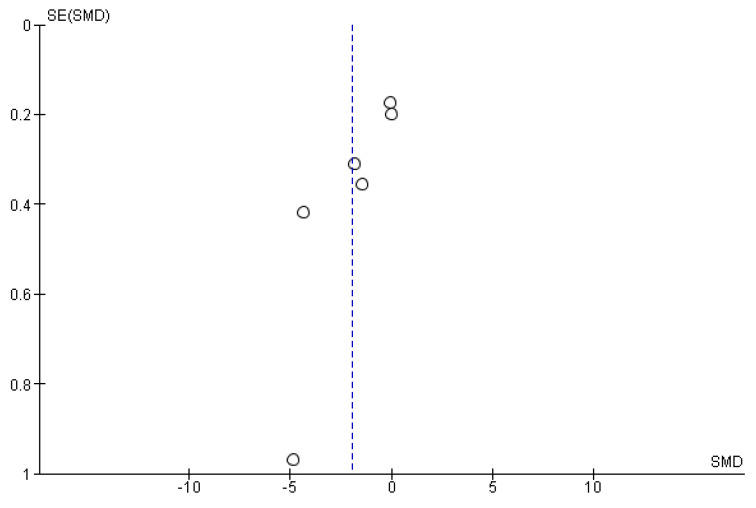
Funnel plot for publication bias assessment.

The meta-analysis, including six out of twelve studies included in synthesis (forest plot reported in Figure 3), demonstrates statistically significant efficacy of the use of essential oils in the reduction of the intensity of pain associated with cancer, assessed through unidimensional pain scales (*p* = 0.002). In particular, the studies contribute almost equally to the results, apart from the study performed by Triana and collaborators [82], which has a lower weight because of the reduced sample size and because it presents wider standard deviation. Moreover, it is possible to highlight high heterogeneity among the studies eligible for the meta-analysis (I^2^ = 96%). The funnel plot suggests risk of publication bias (Figure 4).

## 4. Discussion

Over 80% of patients affected by cancer suffer from pain, which therefore represents one of the most fearsome consequences of cancer [83]. The present systematic review and meta-analysis highlights the paucity of clinical trials in the field of aromatherapy and essential oil use to manage pain associated with cancer. All the studies eligible for inclusion in this analysis include patients aged over 18, apart from the trials conducted by Ndao and collaborators [79] and Triana and coworkers [82] focusing on a pediatric population. However, many cancer types allow longer survival than in the near past; thus, the possibility of age-related comorbidities needs to be taken into account. In particular, apart from the study conducted by Triana and colleagues in which pain is inferred and rated by the nurse [82], the capability of self-reporting of pain and of answering to the assessment of pain intensity through the VAS/NRS/VRS is one of the most common inclusion criteria of the studies retrieved by this systematic search. This aspect is noteworthy since it points at the need for more appropriate pain assessment during cancer. In particular, cancer-related pain is characterized by a multidimensional nature consisting of different physiopathology and etiology and including important sensory, affective, cognitive, and behavioral components, and research still lacks the identification of these fundamental features [84]. Therefore, pain assessment through unidimensional scales, although appropriate for acute conditions and for the evaluation of the sole intensity domain, should be flanked and replaced by use of multidimensional scales as the Brief Pain Inventory (BPI) [85]. In fact, the BPI allows to measure both the sensory dimension of pain intensity and the reactive dimension of interference of pain in the patient’s life [85]. This is increasingly important for the evaluation of the efficacy and safety of essential oils on pain in integrative oncology. The studies found by the present search of databases and inspection of references in the literature demonstrate a main effect of essential oils on general well-being and sleep. However, these symptoms and the benefits of them might be linked to pain processing [86], although not always being detected by an appropriate measure tool, mainly in older patients and in the case of depression [87]. This is supported by meta-analysis that demonstrates the effectiveness of essential oils used as aromatherapy in the reduction of the intensity of cancer-related pain, assessed through unidimensional pain scales (*p* = 0.002). Nevertheless, in agreement with the lack of use of homogeneous and appropriate devices, only six out of twelve studies included in the synthesis are eligible for meta-analysis and this is proven by the high heterogeneity among the studies (I^2^ = 96%) and by the publication bias occurring in the field of essential oils, as well as of oral supplements and nutraceuticals [88,89]. In particular, the included studies underline very different study designs, and some concerns in terms of risk of bias, mainly arising from the inadequate baseline assessment and outcome data and also due to small sample size. For instance, the study performed by Triana and collaborators [82] presents a lower weight in the meta-analysis because of its small sample size. The issue of pain assessment is even more important for aged populations with cognitive decline, needing suitable observational tools [90,91,92], as well as valid and reliable methods with good psychometric and clinimetric properties in this setting, and deserving consideration for additional sources of pain and their treatment [93,94,95,96,97]. In addition, different cancer types can influence pain, thus making the comparison more difficult. One of the most used essential oils is lavender essential oil, but as it involves mainly the cholinergic system, it is not endowed with a strong preclinical rationale for analgesic activity [98,99]; that occurs instead for BEO. The lack of efficacy of the latter in the study by Ndao and collaborators [79] might be, at least in part, explained by the small sample size consisting of patients with different diagnoses and treatment histories. Moreover, the lack of reduction of pain intensity using essential oils in some studies can be due to a late start of treatment which can act only as palliative when chronic pain is established, especially for CIPN, while it should be prevented with earlier therapy [100,101]. Therefore, to provide good certainty of the body of evidence for the management of cancer-related pain using essential oils, it is necessary to establish for natural products a step-by-step preclinical-to-clinical pathway to provide a rationale for effective and safe use.

## Figures and Tables

**Table 1:** Main characteristics of the studies included in the analysis according to the Preferred Reporting Items for Systematic Reviews and Meta-Analyses (PRISMA) 2020 recommendations.

Study	Population	Intervention	Comparator	Outcome Measures	Study Design	Power Analysis	Ethical Committee/ Institutional Review Board Approval and Request for Informed Consent	Results
Blackburn et al., 2017 [71]	Patients aged 18 years or older, having a new diagnosis of acute, myelogenous leukemia, and initiating four weeks of intensive induction chemotherapy	Lavender, peppermint, or chamomile	Placebo	Edmonton Symptom Assessment Scale–Revised with Visual Analog Scale (VAS)	Randomized, cross-over, wash-out trial, N = 50; First Aromatherapy, N = 25; First placebo, N = 28	+	+	Aromatherapy = 2.84 ± 2.23; Placebo = 2.84 ± 2.23
Blackburn et al., 2021 [72]	Women affected by locally advanced cervical cancer	Lavender, lemon, peppermint, and foot reflexology	Standard of- care management medications	Numeric rating scale (NRS)	Randomized, controlled trial, N = 41; Intervention, N = 22; Control, N = 19	+	+	Intervention = 1.5 ± 1.3; Control = 4.4 ± 2.5
Deng et al., 2021 [73]	Women > 18 years old, subjected to breast cancer surgery	Aromatherapy alone or in combination with music therapy	Usual care or music therapy	VAS	Randomized, open-label, controlled trial, N = 160; Aromatherapy, N = 40; Combination therapy, N = 40; Music therapy, N = 40; Usual care, N = 40	Not reported	+	Combination therapy = 2.03 ± 0.83; aromatherapy alone = 3.38 ± 0.90; Usual care = 6.13 ± 1.02
Ha et al., 2022 [74]	Breast cancer patients with taxane acute pain syndrome 20 years of age or older	Aroma lymphatic tressage (Frankincense is used as essential oil and sweet almond oil as a carrier oil) in addition to standard care	Standard care (acetaminophen/tramadol)	VAS	Phase II, randomized, cross-over trial, N = 65. First standard care, N = 22; First aroma lymphatic tressage, N = 33	+	+; Trial registration = KCT0005758	No significant difference in pain score (*p* = 0.368) or toxicity are reported
Ilter et al., 2019 [75]	Patients diagnosed with cancer undergoing port catheter insertion aged 18 years or older	Aromatic mixture prepared by diluting orange, chamomile, and lavender oil at the ratio of 1:1:1 in 70 mL distilled water for inhalation	Routine practices	VAS	Non-randomized, controlled trial, N = 60; Intervention, N = 30; Control, N = 30	+	+	Aromatherapy (5 ± 1.2) reduces pain during and after the procedure in a statistically significant manner in comparison with control (i.e., no treatment; 7.4 ± 1.4)
Izgu et al., 2019 [76]	Patients with Chemotherapy-Induced Peripheral Neuropathic (CIPN) Pain due to oxaliplatin of age of 18 years or older	Aromatherapy massage (peppermint, chamomile, and rosemary blended in 1:1:1 proportion at 1.5% in 50 mL of coconut oil)	Routine care	Douleur Neuropathique 4 Questions	Open-label, quasi-randomized, controlled, pilot study, N = 46; Intervention, N = 22; Control, N = 24	+	+	At week 6, the rate of neuropathic pain is significantly lower in the aromatherapy massage group
Louis and Kowalski, 2022 [77]	Homecare hospice patients with terminal cancer	Aromatherapy with humidified lavender essential oil (3%)	Control (no treatment/water humidification)	VAS	Quasi-experimental, repeated measures, one-group design N = 17	-	+	Not significant difference
Maddocks-Jennings et al., 2009 [78]	Patients aged over 18 affected by radiation-induced mucositis of the oropharyngeal area during treatment for head and neck cancers	Gargle containing 2 drops of a 1:1 mix of the essential oils of manuka and kanuka in water + usual oral care as prescribed	Bottle of sterile water for gargling + usual oral care as prescribed. Instead of placebo, receives usual care	VAS	Randomized, placebo-controlled, feasibility study, N = 19; Active group, N = 9; Placebo group, N = 6; Control group, N = 8	-	+	Within the active group, n = 2 patients experience pain scores ≥ 3, n = 5 from the control group, and n = 4 from the placebo group
Ndao et al., 2012 [79]	Children and adolescents undergoing stem cell infusion	Respiratory administration of bergamot essential oil (BEO) through stream aromatherapy diffuser	Placebo consisting in non-essential-oil-based scented shampoo	VAS	Randomized, placebo-controlled, double-blind trial, N = 37; Intervention, N = 17; Placebo N = 20	+	+	No significant effect on pain
Shammas et al., 2021 [80]	Women (ages 18 to 85 years) subjected to microvascular breast reconstruction due to breast cancer	Lavender oil	Coconut oil	VAS	Prospective, single-blinded, randomized, controlled trial, Intervention, N = 27; Control, N = 22	-	+	No significant differences in the perioperative setting for pain scores (*p* = 0.30)
Soden et al., 2004 [81]	Patients with advanced cancer	Aromatherapy massage with lavender essential oil and an inert carrier oil	Massage with an inert carrier oil alone or no intervention	VAS and Verbal Rating Scale (VRS)	Randomized, controlled trial, N = 42; Aromatherapy massage, N = 16; Massage, N = 13; Control, N = 13	+	+	No statistically significant difference in pain intensity from final to baseline assessment
Triana et al., 2022 [82]	School-age children and adolescents (7–17 years), with a diagnosis of cancer, experiencing chronic pain (for longer than 1 month), and receiving moderate analgesics/opioids	Aromatherapy inhalation of a scent that the participant likes (favorite, aloe vera)	Standard care (painkillers and a relaxing technique)	VAS	Quasi-experimental with a consecutive sampling, N = 20; Intervention group, N = 10; Control group, N = 10	-	+	Inhaled aromatherapy significantly reduces chronic pain (*p* = 0.001) compared with standard care. At minute 30, Intervention = 0.4 ± 0.97 and Control = 6.6 ± 1.43

## Data Availability

The original data presented in the study are included in the article.
